# Doxorubicin-Induced Cardiotoxicity in Collaborative Cross (CC) Mice Recapitulates Individual Cardiotoxicity in Humans

**DOI:** 10.1534/g3.119.400232

**Published:** 2019-07-01

**Authors:** Caroline J. Zeiss, Daniel M. Gatti, Olga Toro-Salazar, Crystal Davis, Cathleen M. Lutz, Francis Spinale, Timothy Stearns, Milena B. Furtado, Gary A. Churchill

**Affiliations:** * Yale University School of Medicine, New Haven, CT 06520,; † The Jackson Laboratory, Bar Harbor, ME 04609,; ‡ Connecticut Children’s Medical Center, University of Connecticut School of Medicine, Hartford, CT 06106, and; § University of South Carolina School of Medicine, Columbia SC 29208

**Keywords:** Anthracyclines, cardiotoxicity, biomarkers, fibrosis, mouse

## Abstract

Anthracyclines cause progressive cardiotoxicity whose ultimate severity is individual to the patient. Genetic determinants contributing to this variation are difficult to study using current mouse models. Our objective was to determine whether a spectrum of anthracycline induced cardiac disease can be elicited across 10 Collaborative Cross mouse strains given the same dose of doxorubicin. Mice from ten distinct strains were given 5 mg/kg of doxorubicin intravenously once weekly for 5 weeks (total 25 mg/kg). Mice were killed at acute or chronic timepoints. Body weight was assessed weekly, followed by terminal complete blood count, pathology and a panel of biomarkers. Linear models were fit to assess effects of treatment, sex, and sex-by-treatment interactions for each timepoint. Impaired growth and cardiac pathology occurred across all strains. Severity of these varied by strain and sex, with greater severity in males. Cardiac troponin I and myosin light chain 3 demonstrated strain- and sex-specific elevations in the acute phase with subsequent decline despite ongoing progression of cardiac disease. Acute phase cardiac troponin I levels predicted the ultimate severity of cardiac pathology poorly, whereas myosin light chain 3 levels predicted the extent of chronic cardiac injury in males. Strain- and sex-dependent renal toxicity was evident. Regenerative anemia manifested during the acute period. We confirm that variable susceptibility to doxorubicin-induced cardiotoxicity observed in humans can be modeled in a panel of CC strains. In addition, we identified a potential predictive biomarker in males. CC strains provide reproducible models to explore mechanisms contributing to individual susceptibility in humans.

The chemotherapeutic use of anthracyclines is limited by their capacity to induce dose-dependent cardiotoxicity (Gianni *et al.* 2008), leading to development of heart failure and death(Gianni *et al.* 2008; Toro-Salazar *et al.* 2015). Efficacy and toxicity of anthracycline agents is highly variable in humans, reflecting the genetic diversity of patient populations as well as gender differences (Franco *et al.* 2011; Lipshultz *et al.* 2015). Multifactorial mechanisms including changes in cellular oxidative stress and viability contribute to acute anthracycline-induced cardiotoxicity (AIC) (De Angelis *et al.* 2010; Simů˚nek *et al.* 2009; Semsei *et al.* 2012). Maladaptive cardiac remodeling, comprising myocyte hypertrophy and extracellular matrix alterations (including loss of normal fibrillar collagen architecture and interstitial fibrosis) are associated with chronic induced toxicity and development of heart failure (Bernaba *et al.* 2010; Toro-Salazar *et al.* 2013; Adamcova *et al.* 2010). Anthracycline induced adverse cardiac remodeling is thought to be mediated through inflammation and cytokine release in response to oxidative stress (Bernaba *et al.* 2010).

Cardiac troponin elevation during chemotherapy is a useful tool in risk stratification for future cardiac injury (Bertinchant *et al.* 2003; Cardinale *et al.* 2004) however, this protein is released in serum only after cardiac tissue damage has occurred. The purpose of this study is to develop a suite of mouse models that model variable susceptibility to cardiotoxicity in human patients. These can be used to interrogate the mechanisms of AIC, and to identify early predictive biomarkers and risk alleles. Current approaches to preclinical testing using animal models aim to minimize variability by limiting genetic diversity. This can yield preclinical findings biased by genetic background and impede subsequent translation to more complex human systems. Genetically diverse mice, such as the Collaborative Cross (CC) better recapitulate the range of phenotypes seen in the human population compared to a single inbred strain (Harrison *et al.* 2014; Collaborative Cross Consortium 2012). CC strains are recombinant inbred mice derived from eight inbred founder strains (A/J, C57BL/6J, 129S1/SvImJ, NOD/ShiLtJ, NZO/HlLtJ, CAST/EiJ, PWK/PhJ & WSB/EiJ) that encompass 90% of the genetic diversity available in the laboratory mouse. CC strains provide reproducible genetic backgrounds with fixed inbred genotypes. Since each CC strain is inbred, each represents a reproducible model with some degree of susceptibility to AIC. Using 10 strains of CC mice, we identify distinct strain and sex-specific susceptibility to AIC. Additionally, we correlate the severity of strain-specific AIC with serum biomarker levels and assess the capacity of these to predict the extent of eventual cardiac injury.

## Methods

### Experimental animals and doxorubicin dosing

**Figure 1:**
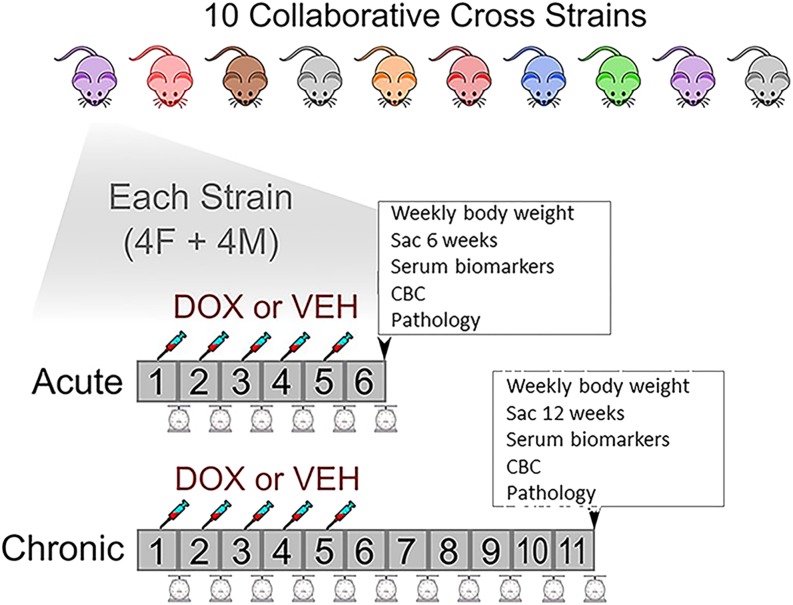
Study design. 10 CC strains were selected. 32 mice per line equally split by sex and treatment regimen (acute and recovery doxorubicin treatment and control groups) were used. A total of 25 mg/kg of DOX (5 tail injections of 5 mg/kg in saline were given at 7-day intervals beginning at 28 days of age. Two time courses for experimental duration were chosen. Eight dosed mice per CC line (4 males and 4 females) were killed one week after the final doxorubicin injection to study acute cardiotoxicity (acute phase). A second cohort of dosed mice (4 males and 4 females) per CC strain were killed 6 weeks after the final DOX injection to study the chronic effects of the drug (chronic or recovery phase). This regimen was duplicated for a similar cohort of control mice, who received weekly injections of saline. Mice were weighed weekly. At either 6 or 12 weeks, acute and chronic cohorts were killed, blood taken for serum biomarkers and complete blood count (CBC), and tissue collected for pathology.

We obtained CC mice from the Jackson Laboratory from each of the following strains: CC001/UncJ, CC010/GeniUncJ, CC011/UncJ, CC019/TauUncJ, CC032/GeniUncJ, CC037/TauUncJ, CC040/TauUncJ, CC041/TauUncJ, CC042/GeniUncJ, and CC051/TauUncJ. For brevity we will drop the substrain designations below. Dose protocols based on a previous juvenile model of doxorubicin-induced cardiac dysfunction were applied(Zhu *et al.* 2008). 32 mice per CC strain equally split by sex and treatment regimen (acute and chronic doxorubicin treatment and control groups) were used. A total of 25 mg/kg of doxorubicin (5 tail vein injections of 5 mg/kg in saline were given at 7-day intervals beginning at 28 days (4 weeks) of age. Two time courses for experimental endpoint were chosen; acute and chronic. Eight dosed mice per CC strain (4 males and 4 females) were killed one week after the final doxorubicin injection to study acute cardiotoxicity (6 weeks; acute phase). A second cohort of dosed mice (4 males and 4 females) per CC strain were killed 6 weeks after the final doxorubicin injection to study the chronic effects of the drug (12 weeks; chronic phase). An equivalent number of control mice (8 males and 8 females) per CC line were subjected to the same experimental regimen, with the exception that they received weekly injections of saline instead of doxorubicin (control). The animals were observed daily for general health, and weighed weekly throughout the course of the study. Animals were group housed by sex in ventilated cages with corn-cob bedding. They were placed on a 12:12 light:dark cycle, and provided regular *ad libidum* chow and water. The Jackson Laboratory (Mount Desert Island, ME) is an AAALAC accredited facility. All procedures were performed in accordance with NIH regulations and approved by the Jackson laboratory Institutional Animal Care and Use Committee under the number 15009. (Figure1)

### Serum biomarker measurement

Blood samples were obtained for each animal at the end of 6 weeks (acute phase) or 12 weeks (chronic phase). Following euthanasia by carbon dioxide asphyxiation, animals were exsanguinated by cardiac puncture and blood collected in serum separator tubes for cardiac troponin I (cTNI), skeletal troponin I (sTNI), myosin light chain 3 (MYL3) and fatty acid binding protein 3 (FABP1) measurement using the Muscle Injury Panel kit from Meso Scale Discovery (MSD; Rockville, MD) following the manufacturer’s instructions. Average values for the lower limit of detection for each assay as provided by the manufacturer (https://www.mesoscale.com/en/products/muscle-injury-panel-3-mouse-kit-k15186c/) are as follows: cTNI (0.0088 ng/ml), FABP3 (0.3 ng/ml), MYL3 (0.048 ng/ml) and sTNI (0.085 ng/ml). 50µl of undiluted serum was used per reaction.

### Complete blood count

An aliquot of blood was collected in Microtainer tubes coated with EDTA (BD Biosciences, Franklin Lakes, NJ) for measurement of white blood cell count (WBC), platelets, red blood cell count (RBC), hemoglobin (Hb), hematocrit (HCT), mean corpuscular volume (MCV), mean corpuscular hemoglobin (MCH) and mean corpuscular hemoglobin concentration (MCHC). Whole blood measurements were performed by the JAX Clinical Assessment Service using the Siemens Advia 120 hematology analyzer.

### Gross necropsy and organ weights

Following euthanasia by carbon dioxide asphyxiation, the pluck was removed, lungs intratracheally inflated with 10% neutral buffered formalin, and the intact heart and lungs, as well as reproductive tract, liver, spleen and right kidney immersed in 10% neutral buffered formalin (NBF) for 7-10 days. Organ weights (kidneys, liver, spleen) were recorded as a percentage of total body weight.

### Histopathology and immunohistochemistry

All tissues underwent routine paraffin processing followed by sectioning at 5 µm and staining with Hematoxylin and Eosin (H&E; Yale Mouse Research Pathology Core in the Section of Comparative Medicine, Yale School of Medicine; http:/mrp.yale.edu). Hearts were sectioned longitudinally to allow visualization of all four chambers. Using hematoxylin and eosin (HE) stained sections, two sections of each heart were scored semi-quantitatively for severity of cardiac injury (adapted from previously published methods (Desai *et al.* 2013; Desai *et al.* 2014; Reagan *et al.* 2013) using three histologic variables. These were cytoplasmic vacuolation, (Desai *et al.* 2014; Ferrans 1978) infiltration by mononuclear cells, (Desai *et al.* 2013) and interstitial fibrosis with myofiber disorganization/atrophy (Ferrans 1978). Each criterion was scored on a 4-point scale with 0 representing normal histologic appearance and 3 representing the most severe score (see S1 Table 1 for scoring system). The scale was applied to each of 5 anatomical regions (left and right atrium, interventricular septum, left and right ventricular free wall). Uncommon histologic findings (mineralization, atrial thrombosis, perivascular lymphocytes) were recorded by region as absent (0) or present (1). The scores for each anatomical region were summed to obtain a total cardiac score for each animal. Two longitudinal renal sections from the right kidney were examined for each animal. Semi-quantitative renal scoring was achieved by recording the approximate percentage per section occupied by a series of glomerular and tubular changes (S1 Table 1). Scores for each change were summed to obtain a composite score for each kidney. A similar approach was used to score pulmonary pathology. Criteria for semi-quantitative scoring of cardiac, renal and pulmonary lesions are given in S1 Table 1. Gonadal, liver and splenic pathology was recorded as present or absent, and described qualitatively only. The pathologist was blinded as to treatment status and strain of each mouse.

The relationship between myofiber degeneration/atrophy, interstitial fibrosis and mononuclear cell infiltration was explored by supplementing H&E staining with reticulin staining to reveal changes in the reticulin (Type III collagen) framework surrounding myofibers, Sirius Red staining (for Type II collagen), and immunohistochemical detection of macrophages (Iba-1; ab107159, 1:100, Abcam, Cambridge, MA), myofibroblasts (alpha smooth muscle actin; SMA; ms113,1:1500, Neomarkers, Fremont, CA), matrix metalloproteinase 2 (MMP-2; 1:100, 6E3F8, ab86607, Abcam, Cambridge, MA) and cleaved caspase-3 (NB100-56113, 1: 1000, Novus Biologicals, Littleton, CO).

### Statistical analysis

i) Body/organ weight: After batch normalization (Johnson *et al.* 2007), we fit linear models to assess effects of treatment, sex, and sex-by-treatment interaction for each timepoint (acute or chronic). Linear models utilize the statistical method of linear regression to describe the relationship between a dependent variable y (or response) as a function of one or more independent variables Xi (or predictors). Generalized linear mixed models include random effects that allow modeling of correlated data that may be non-normally distributed. This allows evaluation of the extent to which interaction between independent variables affects the response. Data were transformed as log(x+c), where the offset constant c was determined by visual inspection of quantile plots to best approximate a normal distribution (Berry 1987). ii) Cardiac and renal scoring: Log(y + 10) transformed raw data were used to fit a linear model that examined the main effects of treatment, sex and timepoint (acute or chronic) with two additional terms examining interactions of treatment with sex and timepoint. iii) Serum biomarkers: Following batch normalization, various transformations were used for cTNI [log(x + 0.001)], FABP3 [log(x)], MYL3 [log(x + 0.01)], and for sTNI [log(x + 0.1)]. Results were reported as estimates (standard error) with Benjamini-Hochberg adjusted p-values. For each strain, a mixed-effects model with sex, treatment, timepoint, sex by treatment and treatment by timepoint as fixed effects and mouse as a random effect was used. This approach was chosen as both acute and chronic measurements for some mice were available, and only acute measurements for others within a strain. To assess whether cTNI and MYL3 levels in the acute phase predict the ultimate extent of cardiac injury (as quantified by cardiac scores in the chronic timepoint), a linear model was applied to each sex. Only mice that survived to the chronic timepoint with provided cTNI or MYL3 measurements during the acute timepoint were included (65 data points, 29 female and 36 males).

### Data access and analysis

**Figure 2:**
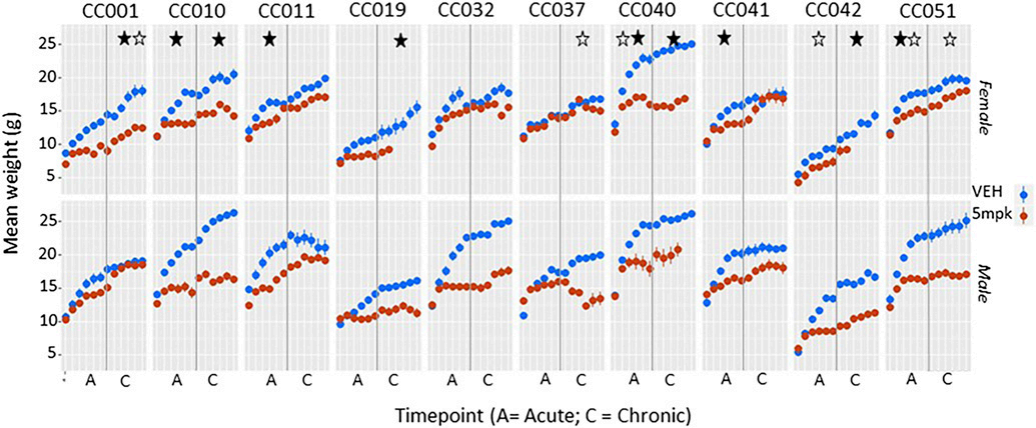
Body weight. Means and standard error at each weekly time point are shown for each strain, with a vertical gray line delineating acute (6 weeks) and chronic (12 week) time points. Females are shown on the top panel and males beneath. Doxorubicin-treated animals (red) all demonstrate impaired growth over time compared to age matched vehicle treated controls (blue). Significant p values (<0.05) for treatment effect (black asterisk), or treatment sex interaction (white asterisk) at acute or chronic timepoints are shown (asterisks are placed on either side of the vertical gray line to indicate significance at either acute or chronic timepoints). CC 40 was a very susceptible strain, with all most animals dying by the end of 12 weeks. A similar trend is seen for females of CC 19 and CC 42.

**Figure 3:**
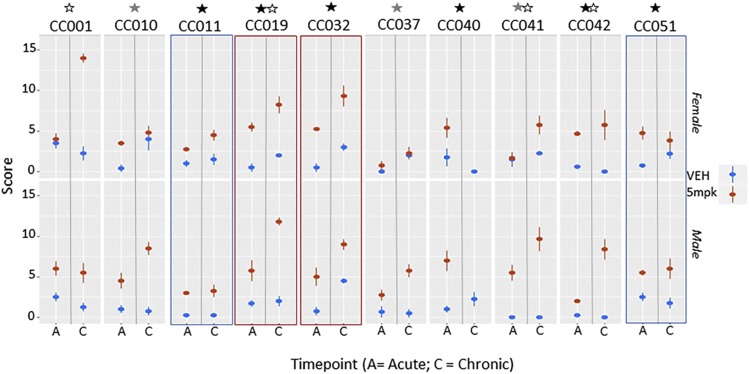
Overview of cardiac scores across all 10 CC lines. Means and standard error at each weekly time point are shown for each strain, with a vertical gray line delineating acute (A; 6 weeks) and chronic (C; 12 week) time points. Females are shown on the top panel and males beneath. The majority of doxorubicin-treated animals (red) demonstrate cardiac injury (increased scores) over time compared to age matched vehicle treated controls (blue). Strains with p values <0.05 for treatment effect, treatment*sex, and treatment*timepoint interactions are indicated with black, gray and white asterisks respectively (located above each strain name). Red and blue rectangles indicate those strains in which both sexes exhibit marked doxorubicin susceptibility or resistance respectively. Strain 40 is very susceptible, with most doxorubicin-treated animals dying before the end of the study.

Supplemental files include S1 Table 1 (semi-quantitative histologic scoring systems), renal pathology and severity scoring (S2 Table 2 and S1 Figure 1), biomarker data for cardiac troponin I, myosin light chain 3, fatty acid binding protein 3 and serum troponin 1 (S3 Table 3, S4 Table), extra-cardiac histopathology (S2 Figure 2) and hematologic data (S3 Figure 3). Supplemental material available at FigShare: https://doi.org/10.25387/g3.8284058.

## Results

### Animal survival and changes in body weight

One control animal in the chronic cardiotoxicity group died prior to completion of the study period. 7 animals across both sexes in the acute treatment group died prematurely (strains CC001, CC 032, CC 042, CC 024, CC 040). 17 animals in the chronic cardiotoxicity group died. Deaths occurred across both sexes and in 7/10 strains (CC 001, CC 032, CC 042, CC 024, CC 019, CC 010, CC 040), but were clearly overrepresented in one strain (CC 040). CC 040 was a very susceptible strain, with most animals dying by the end of 12 weeks. A similar trend was seen for females of CC 019 and CC 042. Consistent with previously reported data across animal species (Bertinchant *et al.* 2003; Desai *et al.* 2013; Manno *et al.* 2016), doxororubicin -treated animals demonstrated impaired growth over time compared to age matched vehicle treated controls. This varied by strain and sex. Significant differences in individual organ weights were noted in four instances (data not shown), but no pattern was identified. (Figure 2)

### Cardiac disease scoring

Scores for each anatomical region were summed to obtain a total cardiac score for each animal. Lesions occurred in all compartments of the heart, with higher scores indicating increasing severity across all anatomical locations. Cardiac severity scores were higher in the majority of doxorubicin-treated animals (Figure 3). These treatment effects reached statistical significance in 6 strains (CC011, CC019, CC032, CC040, CC042, CC051) (Table 1). In CC011, significance was driven by consistently low scores in controls, as scores in treated animals were relatively low at both time points. Thus we did not consider CC011 to be a strain with a strong adverse treatment effect. More severe cardiac pathology was noted in most chronically treated animals, timepoint (acute *vs.* chronic) was statistically significant for strain CC032, and the interaction of treatment with timepoint was significant in four strains (CC001, CC019, CC041, CC042). Susceptibility to doxorubicin-induced cardiac injury varied by sex with greater injury occurring in either males (CC010, CC037, CC041) or in females (CC001). No strain was immune to some degree of cardiac damage, however varying strain susceptibility to cardiac toxicity induced by doxorubicin could be discerned. Overall, three strains appeared relatively resistant to chronic cardiac injury (CC011, CC037 and CC051), with all remaining strains experiencing chronic injury, most severe in CC019 and CC032, CC041 and CC042 (note that most animals in CC040 had died by the chronic stage, and are absent from the analysis). These animals had high renal scores in the acute stage and are likely to have died from combined cardiac and renal toxicity – pathology was not available. Rare vehicle treated animals experienced myofiber vacuolation, disintegration and focal fibrosis - these clustered by strain and were considered a manifestation of mild strain-specific cardiac pathology unrelated to doxorubicin exposure (CC001, CC019, CC041).

**Table 1:** Statistical analysis of semi-quantitative cardiac scoring

Strain	Treatment	Sex	Time	Sex X Trt	Trt X timepoint
CC 01	0.076 (0.041)	−0.034 (0.034)	−0.045 (0.034)	−0.019 (0.048)	0.14 (0.048) *
CC 10	0.067 (0.031)	−0.031 (0.026)	0.052 (0.027)	0.099 (0.036) *	0.021 (0.037)
CC 11	0.072 (0.02) **	−0.039 (0.016)	0.009 (0.016)	0.024 (0.023)	0.021 (0.023)
CC 19	0.138 (0.025) ***	−0.028 (0.02)	0.034 (0.02)	0.015 (0.028)	0.075 (0.029) *
CC 32	0.172 (0.025) ***	−0.029 (0.021)	0.112 (0.021) ***	−0.038 (0.029)	−0.01 (0.029)
CC 37	−0.003 (0.028)	−0.022 (0.023)	0.036 (0.023)	0.114 (0.031) **	0.038 (0.031)
CC 40	0.136 (0.04) **	0.03 (0.033)	−0.01 (0.033)	0.033 (0.052)	0.153 (0.061)
CC 41	0.006 (0.031)	−0.069 (0.03)	0.019 (0.03)	0.178 (0.039) ***	0.097 (0.039) *
CC 42	0.094 (0.032) *	−0.007 (0.024)	−0.018 (0.024)	0.009 (0.036)	0.131 (0.036) **
CC 51	0.103 (0.033) *	−0.022 (0.027)	0.015 (0.027)	0.024 (0.037)	−0.028 (0.037)

Linear model examining main effects (sex, treatment;Trt and time) and two additional treatment interactions (with sex and time). All values are given as estimates (standard error). Benjamini-Hochberg adjusted p-values are indicated as asterisks.

* *P* < 0.05.

** *P* < 0.01.

*** *P* < 0.001.

CC040^†^: all but two animals in the chronic group died prematurely.

### Qualitative cardiac pathology

**Figure 4:**
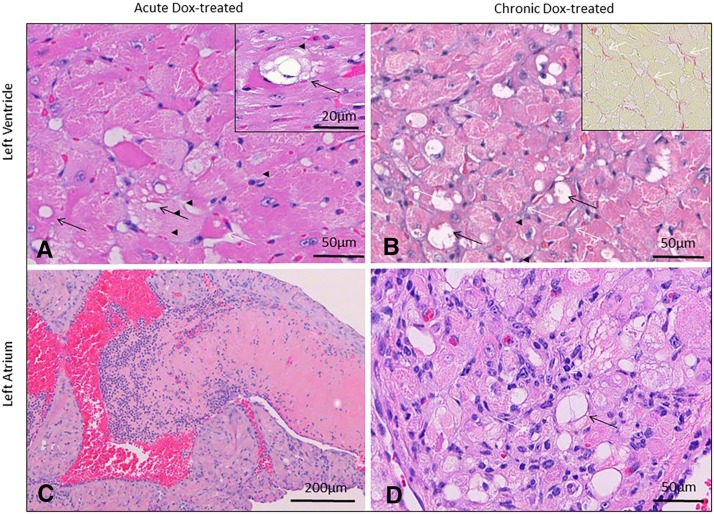
Cardiac histopathology in animals 6 weeks (acute) and 12 weeks (chronic) after initiating doxorubicin (Dox) treatment (25mg/kg total cumulative dose, acute). Intracytoplasmic vacuolation typical of doxorubicin toxicity (black arrows, A and inset), accompanied by rare myofiber atrophy (white arrow, A) prevailed in acutely intoxicated mice. In the chronic phase (B), pathology was dominated by areas of disorganization with marked variation in myofiber diameter, myofiber vacuolation in some animals (black arrows), and fine chicken-wire like fibrosis (white arrows, C and inset, Sirius Red). Atrial pathology was accompanied in some cases by atrial thrombosis (C). Atrial lesions were characterized by myofiber vacuolation (white arrows, D), myofiber atrophy/loss, and lymphohistiocytic inflammation (black arrows, D). Atrial lesions (illustrated in an acute phase animal in C) were more severe in the chronic phase. Hematoxylin and eosin (A, D); Sirius Red staining (inset, B). Bar = 50µm (A, B, inset B, D); 20µm (inset A); 200 µm (C).

**Figure 5:**
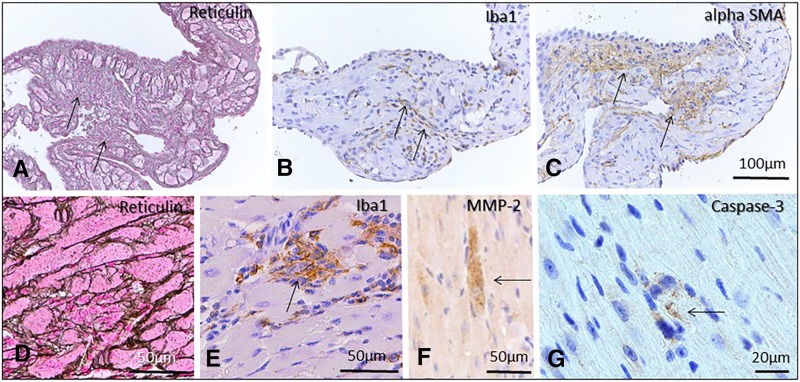
Pathology in DOX treated acute and recovery phase mice. Regions of myofiber loss and frank replacement fibrosis were noted, most commonly in atria (A, acute phase), and rarely in ventricles (D, recovery phase). These areas were accompanied by macrophage infiltration (B, E) and myofibroblast proliferation (C) consistent with fibroplasia. Rare myofibers were matrix metalloproteinase 2 (F, recovery phase animal) or caspase-3 positive (G, acute phase animal). Reticulin staining (A, D); Immunohistochemistry: Iba 1(B, E; macrophages), alpha SMA (C), MMP-2 (F) and cleaved caspase -3 (G) Bar = 100µm (A-C); 50µm (D-F); 20µm (G).

**Figure 6:**
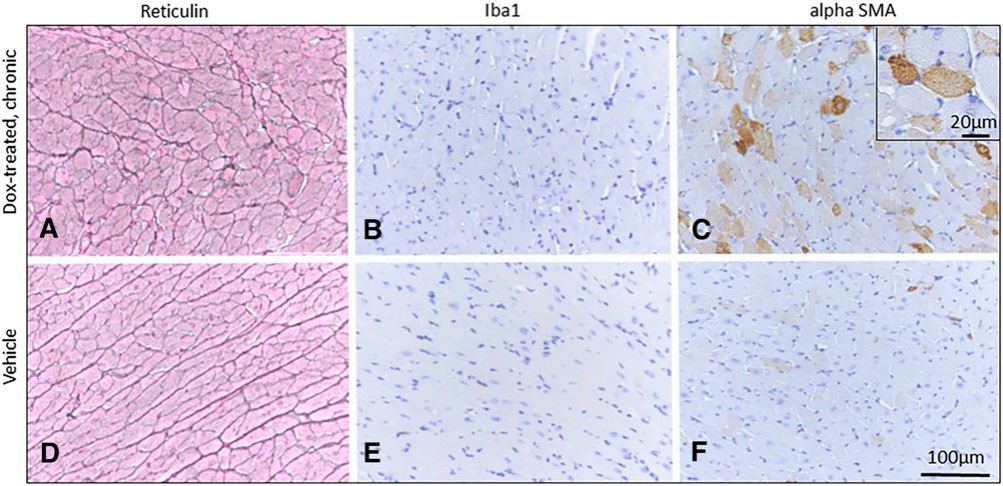
Diffuse interstitial cardiac fibrosis in doxorubicin treated chronic phase mice (left ventricle, A-C) compared to vehicle treated control mice (D-F) at a similar location. Reticulin stains (A) revealed myofiber disorganization, variation in myofiber diameter and fine interstitial fibrosis. This was accompanied by minimal macrophage infiltration (B) and myofibroblast infiltration (C). Instead, SMA expression was increased in myofiber cytoplasm (C, inset). Reticulin staining (A, D); Immunohistochemistry: Iba 1(B, E; macrophages), alpha SMA (C, F). Bar = 100µm (A-F); 20µm (inset, C).

Acutely intoxicated hearts experienced subtle changes characterized primarily by intracytoplasmic myofiber vacuolation typical of doxorubicin toxicity (Figure 4). Additional changes included disintegration of individual myofibers, aggregation of small numbers of macrophages (Figure 5), or regions of subtle disorganization characterized by subjective individual myofiber atrophy. These changes were randomly scattered through the myocardium, but noted most commonly in ventricles and interventricular septum. Atrial pathology was similar in nature, and could be locally more severe in individual animals, with atrial thrombosis (Figure 4), myofiber loss and fibrosis, macrophage and myofibroblast infiltration (Figure 5). Pathology in chronically intoxicated hearts was generally more extensive. The prevailing pattern was one of myofiber disorganization, marked variation in myofiber diameter and fine chicken-wire like interstitial fibrosis (Figure 4, Figure 6). Intracytoplasmic vacuolation did not prevail, but was still evident in some animals. Rarely, focal regions of myofiber loss and frank replacement fibrosis were noted (Figure 5). This pattern was more common in atria and was accompanied by scattered macrophage infiltration and myofibroblast proliferation consistent with fibroplasia (Figure 5)(Zhao *et al.* 2015). In chronically intoxicated animals, histologic abnormalities were often very severe in atria, where they were often accompanied by lymphoplasmacytic and histiocytic inflammation, myofiber loss, focal scarring and atrial thrombosis. As noted in other doxorubicin toxicity studies (Kobayashi *et al.* 2016; Toya *et al.* 2014), macrophage and myofibroblast infiltration were far less prominent in areas of diffuse myofiber disorganization and fine fibrosis (Figure 6) - in these areas, SMA expression was increased in myofiber cytoplasm, consistent with pathological re-expression fetal markers (Szibor *et al.* 2014). Rare individual myofibers expressed Mmp2, most commonly in the chronic phase (Figure 5). Myofibers immunopositive for cleaved caspase-3 were uncommon, even during the acute phase (Figure 5).

### Extracardiac pathology

Renal toxicity was noted in several strains. Renal severity scores tended to be higher in the majority of doxorubicin-treated animals (S1 Figure 1), with significance treatment effects seen in 3 strains (CC011, CC019, CC040) (S2 Table 2). All of these strains also experienced significant cardiotoxicity. Susceptibility to doxorubicin-induced renal injury varied by sex with greater injury occurring in males (CC001, CC037, CC040, CC042). Of these strains, a similar male susceptibility to cardiotoxicity was noted in CC037. Qualitatively, the least severe and most common renal changes were characterized by increased glomerular cellularity. More severe changes involved glomeruli (increased mesangial matrix, segmental tuft atrophy or diffuse glomerulosclerosis), tubules (focal tubular basophilia, tubular loss, atrophy and dilation) and interstitium (fibrosis and mononuclear inflammation; S2 Figure 2). Splenic changes ranged from expansion of red pulp with marked extramedullary hematopoeisis to complete red pulp collapse in both acute and recovery treatment groups (S2 Figure 2). White pulp remained intact, consistent with relatively unchanged lymphocyte counts.

Doxorubicin treatment in males was invariably associated with severe testicular degeneration. Untreated males of some strains experienced mild testicular degeneration of a similar nature, consistent with previously reported strain-dependent testicular pathology (CC001, CC032, CC051, CC010)(Shorter *et al.* 2017).

Pulmonary pathology consistent with heart failure was evident in only one strain (CC040) in recovery phase animals with very high cardiac scores. Striking pulmonary vascular pathology (intense perivascular mixed inflammation with marked endothelial proliferation) was noted in untreated mice of one strain (CC032) consistent with strain–associated pathology unrelated to doxorubicin treatment.

### Serum biomarkers

**Figure 7:**
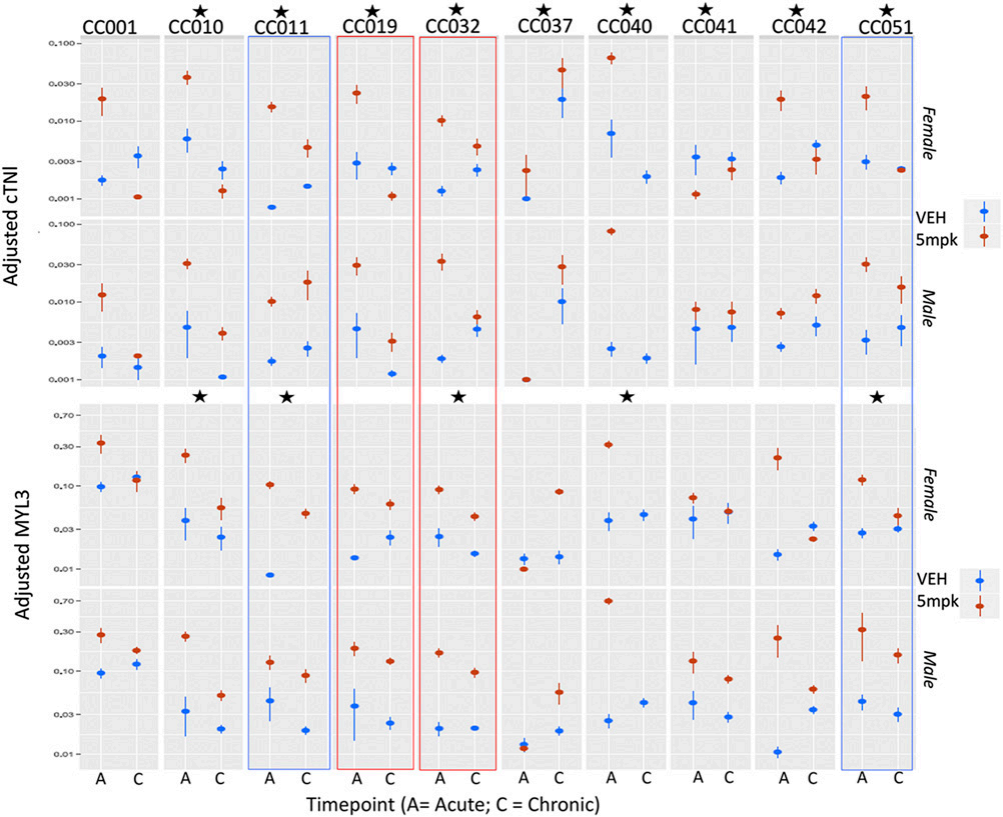
Overview of cTNI and MYL3 levels across all 10 CC strains. Means and standard error at each weekly time point are shown for each strain, with a vertical gray line delineating acute (A; 6 weeks) and chronic (C; 12 week) time points. Females are shown on the top panel and males beneath. The majority of doxorubicin-treated animals (red) demonstrate cardiac injury (increased scores) over time compared to age matched vehicle treated controls (blue). Strains with p values <0.05 for treatment effect are indicated with black asterisks. Additional significant treatment*sex, and treatment*timepoint interactions are shown in Supplementary Tables 3 and 4.

Alteration in serum cardiac troponin I (cTNI), myosin light chain 3 (MYL3), fatty acid binding protein 3 and serum troponin 1 in acute and chronic phases were evaluated (S3 Table 3). Only cTNI (pg/ml) and MYL3 (pg/ml) exhibited significant treatment effects across multiple strains (Figure 7 and S4 Table 4). Both cTNI and MYL3 tended to decrease between acute and chronic phases, in contrast to cardiac scores which increased over time. These data indicate that serum levels of these biomarkers are highest during acute cardiac injury, and decline during the chronic phase, despite ongoing or worsening of cardiac injury. The exception to this was strain CC 37, in which cTNI and MYL3 levels increased over time. cTNI exhibited a significant treatment effect in almost all strains, compared to a similar finding in 5/10 strains for MYL3, indicating that cTNI is the more sensitive indicator of acute cardiac injury. We employed a linear model based test to assess whether troponin levels in the acute phase predict the ultimate extent of cardiac injury (as quantified by cardiac scores in the chronic timepoint). We evaluated each sex separately to allow for potential sex-specific differences. The significance for cTNI was quite low (*P* = 0.42 and *P* = 0.30 for females and males, respectively) indicating that cTNI values in the acute phase do not reliably predict the ultimate extent of cardiac injury. When MYL3 is combined with cTNI, the predictive power remained poor for both biomarkers in females (*P* = 0.14 and *P* = 0.13 for cTNI and MYL3, respectively). However in males, MYL3 appeared to be predictive for extent of ultimate cardiac injury in the chronic phase (*P* = 0.15 and *P* = 0.94 for cTNI and MYL3, respectively).

### Hematologic change

While doxorubicin did not greatly affect white blood cell counts, it had a strong effect on red blood cell counts (RBC). The most consistent hematologic change was anemia that manifested during the acute period (S3 Figure 3). Anemia was regenerative and had recovered in the chronic phase. RBC decreased at the acute time point in almost all strains, except CC051. Most strains recovered by the chronic time point, but some strains (CC001, CC011, CC042) still had decreased RBC. Anemia was accompanied by increased red blood cell distribution width (RDW) in all strains at the acute time point, indicative of a regenerative response, and consistent with extramedullary hematopoeisis noted histologically. Most strains had recovered by the chronic time point, except for CC032 and CC042. Platelets increased at the acute time point across gender (CC011, CC040), or sex-specifically (CC001, CC019, CC032, CC042). Most strains had recovered by the chronic time point. Doxorubicin did not cause myelosuppression at either the acute (orange) or chronic (blue) time points, and white blood cell (WBC) counts were altered in varying patterns. Many strains (CC010, CC040, CC041) showed increased neutrophil counts at the acute time point. This increase persisted at the chronic time point in some strains (CC010, CC032, CC041).

## Discussion

Our approach in this study was to utilize cardiac scoring as the primary outcome measure of cardiac injury, and to assess the capacity of biomarker scores to accurately predict the extent of cardiac injury. Accompanying changes in body weight, extracardiac pathology and hematologic responses were secondary measures. We demonstrated clear strain-dependent variation in cardiac (and renal) susceptibility to doxorubicin induced toxicity. All strains experienced some degree of cardiac injury –this invariably worsened over time in the chronic group despite cessation of doxorubicin treatment by the end of week 6. Extremes of clear high (CC019, CC032 and CC040) and low (CC011 and CC051) susceptibility across both sexes were identified in five strains. In remaining strains, sex-dependent variations in susceptibility were present. Consistent with previous reports(Jenkins *et al.* 2016), males were more severely affected in most strains (CC010, CC019, CC041, CC042, CC051) with females being more severely affected in only one strain (CC001).

All anatomical regions of the heart were histologically affected – lesions were typically scattered and quite rare in the acute phase of the disease. Myofiber vacuolation characteristic of anthracycline toxicity dominated acute cardiac histopathology, however, subtle myofiber disorganization and atrophy presaging later architectural distortion could already be appreciated at this stage(Ferrans 1978). Cleaved caspase-3 positive myofibers were present in the acute phase, however these were rare (0-5/section), indicating that extensive activation of terminal apoptotic pathways was minimal, or alternatively, was short-lived, or accompanied by necrotic forms of cell death (Zhang *et al.* 2016). In chronic phase animals, two patterns of cardiac fibrosis were identified. By far the most common was fine chicken-wire like interstitial fibrosis (containing Sirius red positive Type 1 collagen) accompanied by pervasive myofiber atrophy and disorganization (most easily appreciable using reticulin stains). Significant infiltration of macrophages (Iba-1) and myofibroblasts (alpha-SMA) was not seen in these areas. Additionally, myofiber cytoplasm in fibrotic regions was strongly alpha-SMA immunoreactive, indicating regression to a fetal phenotype (Szibor *et al.* 2014), and raising the possibility that myofibers themselves contribute to pathologic extracellular matrix remodeling through an aberrant transcriptional program (Bowers *et al.* 2010). This notion is underscored by expression of Mmp-2 in myofibers, thus implicating these in collagen remodeling. Interstitial and perivascular deposition of collagen is characteristic of late stages of cardiotoxicity and has been identified in subjects with AIC undergoing cardiac transplantation (Bernaba *et al.* 2010). A second pattern of fibrosis characterized by focal marked myofiber loss, replacement fibrosis (discrete scarring) and dense infiltration by macrophages and myofibroblasts was evident. This was rare in ventricular wall and much more common in atria. Severity of atrial lesions was marked in chronic phase animals, and quite commonly associated with lymphocytic inflammation and atrial thrombosis. The latter is a previously noted lesion in murine (but not human) doxorubicin toxicity(Fujihira *et al.* 1993).

cTNI is considered a myocardium-specific biomarker, whereas MYL3 is present in both cardiac and skeletal muscle (Tonomura *et al.* 2012). Regardless, both were generally elevated in treated acute phase animals, and declined over time in chronic phase mice of all strains except one (CC 037). These results suggest that myofibers are injured by direct cardiotoxicity during periods of doxorubicin administration, but that different mechanisms contribute to adverse cardiac remodeling with development of progressive microscopic fibrosis during the chronic phase. Previous studies have demonstrated that AIC is mediated in part by an effect on the collagen matrix with loss of extracellular matrix and increase in fibrosis(Bernaba *et al.* 2010; Caulfield and Bittner 1988; Toro-Salazar *et al.* 2013). Activation of matrix metalloproteases (MMPs) and decreased levels of tissue inhibitors of MMPs (TIMPs)(Kong *et al.* 2014) and inflammatory mediators play an important role in LV remodeling. Myocyte hypertrophy (Spinale and Zile 2013; Spinale 2002; Yokoyama *et al.* 1997), myosin heavy-chain switch(Kubota *et al.* 1997), cardiac myocyte apoptosis (Krown *et al.* 1996) and extracellular matrix alterations with early loss of fibrillar collagen followed by late microscopic fibrosis, contribute to adverse cardiac remodeling ultimately leading to heart failure (Liew and Dzau 2004; Kong *et al.* 2014). In our pediatric early AIC cohort, we have identified biomarker signatures in inflammatory, structural and cell growth/viability pathways (Toro-Salazar *et al.* 2018). cTNI and MYL3 exhibited significant treatment effects across 9 and 5 strains respectively, suggesting that cTNI was the more sensitive indicator of acute injury. In agreement with previous reports (Cardinale *et al.* 2004; Bertinchant *et al.* 2003; Reagan *et al.* 2013), cTNI elevation in the acute phase was not predictive of the eventual extent of cardiac injury as assessed by cardiac score in either sex (Armenian *et al.* 2014; Moazeni *et al.* 2017). MYL3 was similarly non-predictive in females, however in males, MYL3 levels in the acute phase were correlated with the extent of eventual cardiac injury, implicating a sex-specific use for this biomarker. The latter finding should be interpreted with caution – MYL3 is not cardiac-specific, and in rodents, its clearance can be influenced by renal toxicity(Tonomura *et al.* 2012).

Both carbon dioxide euthanasia and cardiac puncture could conceivably alter troponin levels in our cohort (Marquardt *et al.* 2018; Everds 2017), however the of cardiac troponins to accurately predict the extent of chronic cardiac injury remains an imperfect science. cTNI is utilized in clinical practice (Horacek *et al.* 2008; Jones *et al.* 2017; Simões *et al.* 2018; Ma *et al.* 2019). Cardiac troponin T has been used to monitor anthracycline induced injury in clinical patients (Blaes *et al.* 2015; Bisoc *et al.* 2019), although its elevation following anthracycline treatment is not invariable (Horacek *et al.* 2008; Lipshultz *et al.* 2004). Both cTNI and cTNT are more reliable indicators of injury immediately following anthracycline exposure (Simões R *et al.*, 2018). Assessing the capacity of cTNT to predict the extent of chronic cardiac injury would be a useful addition in subsequent studies using murine model systems(Jenkins *et al.* 2016). Future studies in susceptible and resistant CC strains will help define regulatory molecular pathways related to AIC predictors in these genetic variants.

Marked renal toxicity was evident in some strains. Lesions recapitulated previously described pathology centered on glomeruli (Yasuda *et al.* 2010) with eventual extension to tubular and interstitial components. In general, renal toxicity was less prevalent than cardiotoxicity. In contrast to cardiac pathology, overall worsening of renal pathology over time was not nearly as marked. While some strains developed both cardiac and renal injury across both sexes (CC019, CC040), others exhibited relatively greater renal toxicity (CC051) indicating that some genetic determinants of these two organ responses are shared, whereas others are independently inherited. Like cardiotoxicity, susceptibility to renal toxicity varied across strain, sex and timepoint. Renal toxicity is well-described in rodents (Lee and Harris 2011) and pigs (Manno *et al.* 2016), but is less common in humans (Bárdi *et al.* 2007). The potential of this lesion to affect biomarker clearance in mice (Tonomura *et al.* 2012), and thus confound translation of biomarker data from mice to humans should not be underestimated. As in other species,(Manno *et al.* 2016) severe testicular degeneration occurred in all treated males, making this lesion the most consistent of all organ responses. Ovarian atrophy was noted in some treated females, however this organ was not available in sufficient sections to adequately assess strain susceptibility of this response.

Doxorubicin treatment generally impeded the rate of weight gain across strains, however the extent of this could not be clearly correlated with the extent of cardiac injury. Because humans receiving doxorubicin have the added variable of neoplasia, and because their weight is very individual, body weight was not considered a translationally useful biomarker. Bone marrow suppression is commonly a dose limiting toxic effect during active doxorubicin treatment in humans (Panis *et al.* 2012), dogs (Ahaus *et al.* 2000) and pigs (Manno *et al.* 2016) . This effect can manifest across erythroid, granulocytic and thrombocytic lineages (Panis *et al.* 2012). We did not observe myelosuppression in our mice. Consistent with previous reports in mice (Desai *et al.* 2013), the most profound hematologic abnormality was anemia during the acute phase. This was regenerative, and red cell counts had normalized by the chronic phase. Marked splenic extramedullary hematopoeisis followed by eventual red pup exhaustion was noted in many doxorubicin treated mice, consistent with marked splenic hematopoeitic compensation for bone marrow toxicity. In contrast to humans, in which splenic extramedullary hematopoiesis is negligible, the adult murine spleen (and to lesser extent the liver) are capable of profound extramedullary hematopoiesis when stimulated (Sonoda and Sasaki 2012).

In this study, a spectrum of cardiac pathology could be demonstrated that exhibited a wide range across strains, but was consistent within strain. Resistant and susceptible strains provide a powerful tool to investigate pathophysiologic mechanisms driving cardiac injury, particularly those that follow acute intoxication to create chronic ongoing injury. These strains are similarly useful in the search for early stage biomarkers that can be used to predict the extent of chronic injury. Because mice within each CC strain are virtually genetically identical (over 90% homozygous), CC mice are most ideally suited to identification of reproducible anthracycline sensitive and resistant phenotypes. These can be used to interrogate cellular mechanisms of AIC, particularly those underlying progressive interstitial fibrosis, and to identify early predictive biomarkers. For the identification of genetic determinants contributing to AIC, genetically heterozygous mice generated from the same 8 parental strains, *i.e.*, the Diversity Outbred (DO) mice, are better suited.
